# Supplementary material for: Hybrid Models and Biological Model Reduction with PyDSTool
Source: PLoS Comput Biol. 2012 Aug 9;8(8):e1002628. doi: 10.1371/journal.pcbi.1002628 (PMC3415397; doi:10.1371/journal.pcbi.1002628)
Supplement: Text S4 — Complete source code for the PyDSTool package (version 0.88.120504). Includes API documentation and help files linking to web pages. This file is identical to the current public release on Sourceforge.net. (ZIP) [file pcbi.1002628.s004.zip › PyDSTool/html/toc-cPickle-module.html]

xml version="1.0" encoding="ascii"?


cPickle


# Module cPickle

---

## Classes

BadPickleGet  
 PickleError  
 PicklingError  
 UnpickleableError  
 UnpicklingError  

## Functions

Pickler  
 Unpickler  
 dump  
 dumps  
 load  
 loads  

## Variables

HIGHEST\_PROTOCOL  
 compatible\_formats  
 format\_version  

---

[hide private]
